# DNAJB13, a type II HSP40 family member, localizes to the spermatids and spermatozoa during mouse spermatogenesis

**DOI:** 10.1186/s12861-014-0038-5

**Published:** 2014-09-19

**Authors:** Weina Li, Gang Liu

**Affiliations:** 1 Institute of Reproduction and Stem Cell Engineering, Central South University, Reproductive and Genetic Hospital of CITIC-Xiangya, Changsha 410078, China

**Keywords:** Dnajb13, Spermatid, Flagella, Spermiogenesis, Motility

## Abstract

**Background:**

Hundreds of HSP40s derived from various species have been identified, of which several proteins are involved in spermatogenesis. DNAJB13 is a type II HSP40/DnaJ protein. In a previous study, we cloned mouse *Dnajb13*, which is up-regulated in cryptorchidism. To date, however, little is known about the localization and functions of DNAJB13 during spermatogenesis. This study intends to identify the expression pattern of DNAJB13 during mammalian spermatogenesis.

**Results:**

RT-PCR and western blot revealed that the *Dnajb13* gene and DNAJB13 protein were expressed not only in the testis but also in several other ciliated cell-containing tissues like the trachea, lung and oviduct. Quantitative PCR showed that the expression of *Dnajb13* mRNA in testis was detectable as early as postnatal week 1, and sharply increased from postnatal week 3. Western blotting and immunohistochemistry determined that the DNAJB13 protein, which was located in the cytoplasm of spermatids and the sperm flagellum, was detectable from postnatal week 4.

**Conclusions:**

Based on the spatiotemporal expression observed in the cytoplasm of spermatids and sperm flagella, we suggest that DNAJB13 participates in spermiogenesis and the motility of mature spermatozoa.

## 1 Background

Spermatogenesis is a complex biological process involving a number of cellular events including mitosis, meiosis, cell migration, apoptosis and differentiation, which enable the germ cells to undergo several developmental stages, from spermatogonia to primary and secondary spermatocytes, round spermatids and eventually spermatozoa. It takes approximately 35 days in mice.

To date, hundreds of HSP40s derived from various species have been identified, of which several proteins are involved in spermatogenesis [1]. A human DnaJ homologue, Dnaja1 (Hsj2), binds to the pituitary tumor-transforming gene protein and then plays a role in spermatogenesis [2]. Evidence from Dnaja1-mutant mice has shown that Dnaja1 plays a critical role in spermiogenesis by regulating androgen receptor signaling. The Dnajb3 (Msj-1) gene encodes a DnaJ protein highly expressed in spermatids and spermatozoa of both rodents and amphibians [3],[4]. It may be involved in vesicle fusion and protein quality control by means of interaction with heat shock proteins [5]. Molecular chaperones belonging to the superfamily of heat shock proteins (HSPs), their co-chaperones, and the ubiquitination/deubiquitination system may be involved in the production of high-quality spermatozoa [6]. HSP40s generally function as co-chaperones in the HSP70 chaperone systems [7]-[10] that are involved in many processes in cells.

Mammalian cells possess a large and diverse family of HSP40/ DnaJ proteins, and they can be divided into three subgroups, type I, II, and III, according to the presence or absence of the Gly/ Phe-rich region and the cysteine repeats [11]. DNAJB13 (*Mus musculus*) (accession number: NP_705755) is a type II HSP40/DnaJ protein. DNAJB13 (*Homo sapiens*) is also known as TSARG6 (testis spermatogenesis apoptosis-related protein 6) [12]. In a previous study, we first reported that DNAJB13 was expressed abundantly in mouse testis and up-regulated in cryptorchidism [13]. Guan et al. characterized DNAJB13 in mature mouse testes and epididymal spermatozoa [14]. To date, however, little is known about the time of origin and location of DNAJB13 during mouse spermatogenesis, which is an important clue to clarifying its function. This study intends to identify the expression pattern of DNAJB13 progressively and constitutively during mammalian spermatogenesis.

## 2 Results and discussion

### 2.1 *Dnajb13* mRNA and DNAJB13 protein strongly expressed in testis, lung, oviduct and trachea

**Figure 1:**
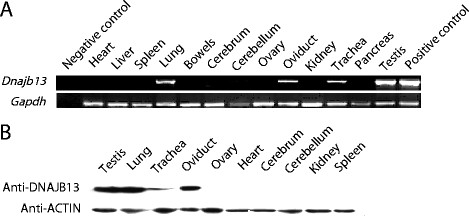
***Dnajb13*mRNA and DNAJB13 protein in different mouse tissues. (A)** Mouse multi-tissue RT-PCR of *Dnajb13*. Total RNA samples extracted from different mouse tissues were subjected to RT-PCR with a *Dnajb13* primer set. The housekeeping gene *Gapdh* was also amplified as a control. In the negative control, the template is double-distilled water. In the positive control, the template is recombinant plasmid pQE/*Dnajb13*. **(B)** Mouse multi-tissue WB of DNAJB13. The beta-ACTIN was also detected as a control.

We performed reverse transcription polymerase chain reaction (RT-PCR) to study the expression profile of *Dnajb13* mRNA in different mouse tissues. The results showed that the *Dnajb13* gene was extremely strongly expressed in adult testis, lung, oviduct and trachea (Figure 1A). *Gapdh* was expressed in all kinds of tissues. We also performed immunoblotting to study the expression pattern of DNAJB13 in different mouse tissues. The results showed that DNAJB13 was expressed in adult testis, lung, oviduct, and trachea (Figure 1B), being consistent with the RT-PCR results. By multi-tissue RT-PCR and western blot, we found that the *Dnajb13* gene and DNAJB13 protein were expressed not only in the testis, but also in several other ciliated cell-containing tissues like the trachea, lung and oviduct. In mammals, both sperm flagella and cilia are related to cell migration and movement. Therefore, we speculated that the *Dnajb13* gene may be associated with cell migration and movement.

### 2.2 Expression pattern of *Dnajb13* mRNA and DNAJB13 protein located at different developmental periods of mouse testis

**Figure 2:**
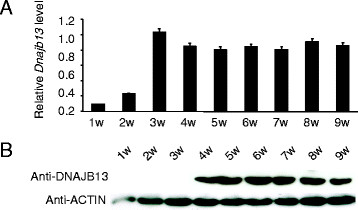
***Dnajb13*mRNA and DNAJB13 protein at different developmental periods of mouse testis. (A)** Quantitative RT-PCR for *Dnajb13* from different periods of mouse testis. The expression of *Dnajb13* mRNA was detected as early as postnatal week 1, and developmentally up-regulated to postnatal week 3, then was constant. After postnatal week 4, its expression was constant at a high level. Each bar of the histogram represents the mean (±SEM) of at least three experiments. **(B)** WB of DNAJB13 from different periods of mouse testis. The beta-ACTIN was also detected as a control. DNAJB13 protein was undetectable until postnatal week 4. There was an apparent delay in DNAJB13 protein translation.

The different developmental periods of 1- to 9-week-old mice testes were collected for total RNA and protein preparations, which were subsequently subjected to real-time quantitative PCR (qPCR) (Figure 2A) and immunoblotting (Figure 2B) analyses, respectively. As shown in Figure 2A, in mouse testis, the expression of *Dnajb13* mRNA was detected as early as postnatal week 1, was developmentally up-regulated at postnatal week 3 when the round spermatids began to appear, and then was constant. After postnatal week 4 its expression was constant at a high level. *Gapdh* was expressed at various development periods in mouse testis as a control. We also performed immunoblotting to study the expression pattern of DNAJB13 in these testes. The expression of DNAJB13 was undetectable until postnatal week 4 when elongated spermatids were apparent (Figure 2B). These data demonstrate that the expression of the *Dnajb13* gene was developmentally regulated and there was an apparent delay in DNAJB13 protein translation. DNAJB13 expressed from postnatal week 4 of mouse testis suggested that DNAJB13 was expressed in the spermatids and spermatozoa.

### 2.3 In situ hybridization of *Dnajb13* in mouse testis at different developmental periods

**Figure 3:**
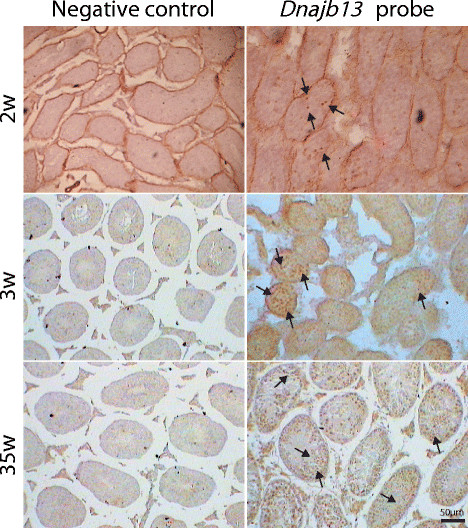
**The result of ISH in mouse testis at various developmental periods.** Germinal cell localization of *Dnajb13* in mouse testis detected by in situ hybridization. In situ hybridization of mouse testis sections, negative control (left) or *Dnajb13* probe (right) DIG-labeled oligonucleotide probes specific for *Dnajb13*. Signals indicating the presence of *Dnajb13* transcript were confined to the seminiferous tubules in association with spermatocytes and round spermatids. The arrow indicates the positive signals. The scale bar represents 50 μm.

The cellular localization of *Dnajb13* mRNA expression was determined by in situ hybridization of mouse testis sections. The specific tawny hybridization signal in the cytoplasm was distributed inside the tubules from week 2 (Figure 3), increased in week 3, and then had a stable expression. Such a distribution is characteristic of the stage-specific expression of *Dnajb13* during mouse spermatogenesis, and is compatible with the qPCR of *Dnajb13* in the mouse seminiferous tubule. The positive signals correspond to the cytoplasm of spermatocytes and round spermatids located along the spermatogonia in the basement membrane distant from the boundary of the tubules. Under identical conditions, the control probe did not give any signal.

### 2.4 Immunohistochemical detection of DNAJB13 in mouse testis at different developmental periods

The seminiferous epithelium from different developmental periods of mouse testis contained different spermatogenic cells. There were only primitive type A spermatogonia in the seminiferous epithelium of 0-week-old and 1-week-old mice. Type A and type B spermatogonia appeared in the seminiferous epithelium of 8-day-old mouse testis. Preleptotene spermatocytes were the smallest germ cells in sections of 10-day-old mouse testis. Pachytene spermatocytes were observed in the testis of 14-day-old mice. Round spermatids began to appear in the seminiferous epithelium of 18-day-old mouse testis [15].

**Figure 4:**
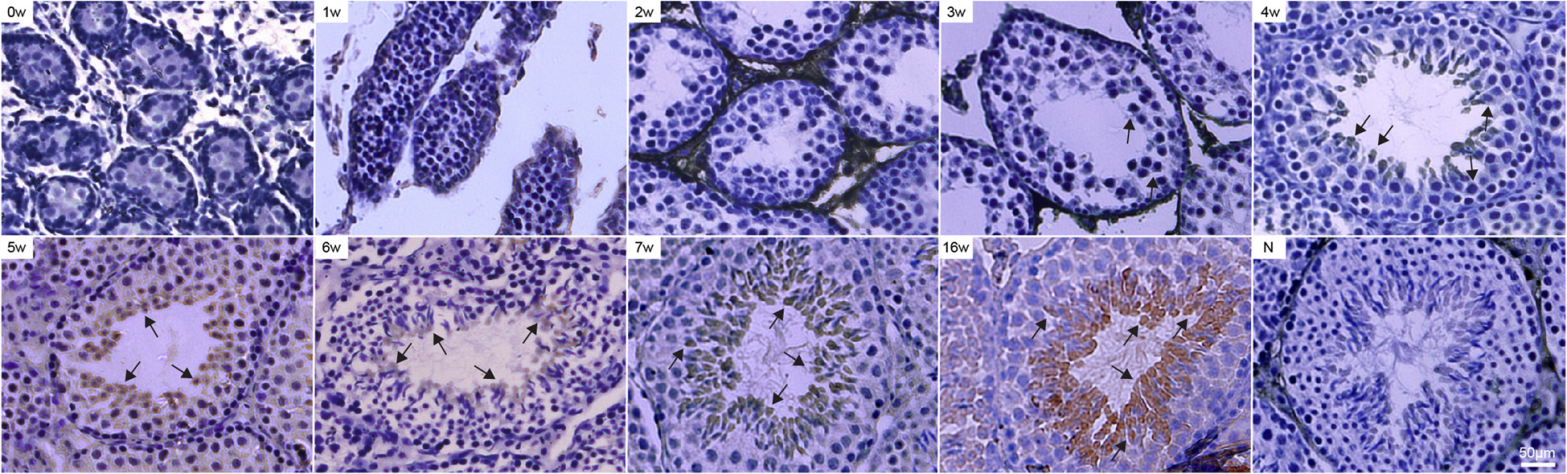
**Immunohistochemical localization of DNAJB13 in mouse testes.** Histological sections were made from testes of weeks 0, 1, 2, 3, 4, 5, 6, 7 and 16 mice. The results showed that DNAJB13 was present in the cytoplasm of spermatids and the flagella of mature spermatozoa. In the negative control stained with goat pre-immune sera, no positive immunosignal was seen. The arrow indicates the positive signals. Scale bar = 50 μm.

Subcellular localization and distribution of the DNAJB13 protein at defined stages of spermatogenesis were revealed. The DNAJB13 immunohistochemical signal was first seen very weakly in the round spermatid cytoplasm of 3-week-old mice; the signal intensity of DNAJB13 increased as spermiogenesis proceeded. A distinct expression pattern of DNAJB13 was restricted to the spermatid layer in the mice from week 4. The DNAJB13 immunosignal was present in the cytoplasm of spermatids and flagella of mature spermatozoa that were about to be released into the tubular lumen (Figure 4), consistent with the WB result of DNAJB13 in different developmental periods of mouse testis. No immunosignal was detected in any other spermatogenic or somatic cells in the testicular sections. The negative control using pre-immune serum was free of any signal.

Notably, there is a discrepancy between the immunoblotting and immunohistochemical results. The WB data indicated that the expression of DNAJB13 was undetectable until postnatal week 4. The DNAJB13 immunohistochemical signal was first seen very weakly in 3-week-old mice, the signal intensity increasing in postnatal week 4. Although round spermatids began to appear in 18-day-old mouse testis, they comprised only 1% of the seminiferous epithelium cells (degenerating and unidentified non-spermatogenic cells have not been included in these data), rising to 4% in 20-day-old mouse testis. Immunoblotting did not detect DNAJB13 protein in postnatal week 3 mice. However, a very weak immunohistochemical signal was observed in cross sections of seminiferous epithelium in 3-week-old mice.

As shown above, DNAJB13 proteins were expressed in testis, trachea, lung and oviduct. We chose testis as a target tissue to investigate the function of DNAJB13 in spermatogenesis. The results of the sequential expression profile in mouse testis showed that the DNAJB13 protein was expressed distinctly at postnatal week 4. More importantly, the positioning study confirmed the expression of DNAJB13 in the cytoplasm of spermatids and the flagella of mature spermatozoa.

The location of DNAJB13 in the flagella of mature spermatozoa indicates its function in sperm motility. The flagellum is a major part of the spermatozoon. The core of the flagellum is a microtubular structure called the axoneme. During spermiogenesis, the axoneme develops from one of the two centrioles to form the flagellum. As the axoneme grows, accessory components including mitochondria, outer dense fibers, a fibrous sheath and an annulus are added to the flagellum to form the sperm middle, principal and end pieces.

In a previous study, Yang C found that RSP16, a homologous protein of DNAJB13 in *Chlamydomonas*, may function as a co-chaperone in the assembly of radial spokes. In a further study, knockdown of RSP16 influenced flagellar motility, and this motility defect could be rescued by RSP16 without the J domain [16],[17]. Guan J considered that DNAJB13 was localized to the radial spokes of the mouse axoneme [18]. Using immunoelectron microscopy analyses of human sperm, we have found that DNAJB13 is not only localized on the radial spokes of the axoneme in human sperm flagella, but also in other flagellar structures (unpublished data). Our results validate the views of Yang C and Guan J. Therefore, we suggest that DNAJB13 influences sperm motility.

The expression of DNAJB13 in spermatids indicates the protein may participate in spermiogenesis. Spermiogenesis is the final stage of spermatogenesis. During this morphogenetic process, shaping and condensation of the nucleus as well as the formation of the acrosome and the tail take place. Spermiogenesis takes about 2–3 weeks in mice. At this stage, the haploid germ cells do not divide, but morphogenesis occurs [19]. These dramatic changes require a stringent, well-coordinated and unique system that regulates specific patterns of gene and protein expression [20]. Several Hsp40 proteins are involved in spermiogenesis. DnaJ protein MSJ-1 (mouse sperm cell-specific DnaJ first homologue) is a molecular chaperone expressed in spermatids and spermatozoa [21]. mUBPy and MSJ-1 label the cytoplasmic side of the acrosomal membrane and the centrosome in mouse spermatozoa, and could have a key role during mouse fertilization [22]. DjA1(−/−) mice revealed a primary defect of Sertoli cells in maintaining spermiogenesis at steps 8 and 9 [23]. More thorough work is needed to elucidate the function and mechanism of DNAJB13 in spermiogenesis.

## 3 Conclusions

In this study, we characterized the profile of the *Dnajb13* gene and DNAJB13 protein in different mouse tissues at different developmental periods. We revealed a spatiotemporal association of DNAJB13, a type II HSP40, with the testis and elucidated that DNAJB13 is expressed in the cytoplasm of spermatids and the flagella of mature spermatozoa. We suggest that DNAJB13 participates in the process of spermiogenesis and sperm movement.

## 4 Methods

### 4.1 Animals

BALB/c mice were purchased from Shanghai, China. Animals were housed in a standard animal facility under controlled temperature (22°C) and photoperiod (12 hs light, 12 hs dark) with free access to water and mouse chow. All animal experiments were approved by the Ethics Committee of Citic-Xiangya Reproductive and Genetic Hospital.

### 4.2 RNA isolation and RT-PCR

The multi-tissues collected from adult BALB/c mice were submerged in Trizol (Invitrogen) immediately to stabilize the RNA. Testes collected from mice at postnatal weeks 1, 2, 3, 4, 5, 6, 7, 8 and 9 were submerged in Trizol (Invitrogen) immediately to stabilize the RNA. We treated RNA samples with DNase I (RNase-free) (NEB) to ensure the elimination of any genomic DNA contamination, then verified the purity of the RNA samples by means of electrophoresis and measuring OD values with a NANO DROP 1000 spectrophotometer (Thermo Scientific) operated by ND-1000 V3.8.1 software. The RNA samples were quantified and the first-strand cDNA was synthesized using a RevertAid™ First Strand cDNA Synthesis Kit (Fermentas). The cDNA samples were subsequently subjected to RT-PCR with *Dnajb13* (951 bp) and *Gapdh* primers (708 bp). PCR was carried out with Pfu DNA Polymerase (recombinant) (Fermentas). *Gapdh* was also amplified with an equivalent amount of cDNA template as a control. For the primer sequences, see Table 1.

**Table 1:** Primer sequences

**Accession number**	**Primer name**	**Sequence**	**Size**	**Annealing**
NM_153527.2	*Dnajb13*-forward	5′ ATGGGGCTGGATTACTAT 3′	951	58°C
	*Dnajb13*-reverse	5′ TTAGGTCAGCAATGCCTG 3′		
NM_008084.3	*Gapdh*-forward	5′ TGAAGGTCGGTGTGAACGGATTTG 3′	708	58°C
	*Gapdh*-reverse	5′ CATTGGGGGTAGGAACACGGAAGG 3′		
NM_153527.2	*Dnajb13*-for real-time forward	5′ AACTTGCCCTGAAGAACCACC 3′	136	58°C
	*Dnajb13*-for real-time forward	5′ CCTTCCTCACCAAACTTGTCAT 3′		
NM_008084.3	*Gapdh*-for real-time forward	5′ GGTTGTCTCCTGCGACTTCAACAGC 3′	231	58°C
	*Gapdh*-for real-time reverse	5′ CGAGTTGGGATAGGGCCTCTCTTGC 3′		

### 4.3 Real-time quantitative PCR (qPCR)

The cDNA synthesis was performed at 37°C for 60 min after heat inactivation at 95°C for 10 min. PCR was performed using 1 × SYBR Green PCR Master Mix (Applied Biosystems, Lab India, Chennai, India) on a real-time PCR system (Prism 7300; Applied Biosystems). Cycling conditions were as follows: 2 min at 50°C, 10 min at 95°C, and 40 cycles of 15 s at 58°C, plus 1 min at 60°C. Commercial software (SDS ver.2.3; Applied Biosystems) was used to calculate ΔΔCt relative expression values for all the genes studied, normalized to the *Gapdh* endogenous control. For the primer sequences, see Table 1.

### 4.4 In situ hybridization (ISH)

We used gene expression images measured using in situ hybridization (ISH). To measure the expression of a target gene, ISH uses DIG-labeled DNA sequences that are complementary to the target gene *Dnajb13* RNA. These DNA probes are cloned and applied to each mouse testis section. The complementary probes hybridize to the target RNA sequence inside the cells, while the non-bound probes are washed away. *Dnajb13* probe sequences: 5′-CTCCATGGACAACTGGCTACGTCTTCCATG-3′, 5-CACCCTCGCTTCCGCAGGGAGCATGACAAC-3′, 5′-TTCATCTTCTTTGACATCCAGTTCCCCACC-3′. This DIG labeling captures the spatial pattern of expression of a target gene across the testis. Preparation of riboprobe and ISH Digoxigenin-11-UTP-labeled riboprobe (DIG-riboprobe; final concentration: 1 μg/ml or 100 ng probe/slide) was used according to the manufacturer’s instructions (Roche Diagnostic). Sections (5–6 μm) were deparaffinized and rehydrated, washed in PBS-T (1 M Na_2_HPO4, 1 M NaH_2_PO4, 1.5 M NaCl, pH 7.4 containing 0.1% Tween 20) and digested with proteinase K (Sigma; 1 μl/ml in PBS-T). The reaction was then blocked with a stop-solution containing 2 mg/ml glycine in PBS-T. After two washes with PBS-T, the sections were post-fixed with 4% formaldehyde in PBS-T for 30 min. Pre-hybridization with hybridization solution consisting of 50% formamide, 50 μg/ml heparin, 500 μg/ml yeast tRNA, 0.1% Tween 20, and 5× standard sodium citrate (SSC: 0.15 M NaCl/0.05 M sodium citrate, pH 7) was carried out for 1 h at 37°C. Hybridization was performed with 15% riboprobe in hybridization solution overnight at 37°C. The sections were rinsed with PBS-T and washing solution (0.3% 20× SSC, 1% Tween 20, in distilled water), incubated at room temperature (r.t.) with horse serum (2% in PBS-T) and denatured at 95°C for 5 min by applying the probe solution to them and hybridized with anti-digoxigenin-Fab-antibody (Roche; diluted 1:100 in the horse serum solution) for 1 h at r.t. Finally, the sections were incubated in a 5-bromo-4-chloro-3-indolyl phosphate/nitro blue tetrazolium liquid-substrate system (BCIP/NBT, Sigma). Control experiments were performed using the corresponding sense cRNA (1 μg/ml). Two specimens from each developmental stage were examined.

### 4.5 Protein preparation

The multi-tissues collected from adult BALB/c mice were lysed in RIPA buffer (150 mM NaCl, 1 mM EDTA, 1% NP-40, 0.5% Na-deoxycholate, 0.1% SDS, 50 mM Tris–HCl, pH 7.6) supplemented with 1 × protease inhibitor cocktail (Roche) in a dounce homogenizer. Testes collected from mice at postnatal weeks 1, 2, 3, 4, 5, 6, 7, 8 and 9 were lysed in RIPA buffer. Lysates were sonicated to disrupt genomic DNA and then centrifuged at 10,000 g for 10 min at 4°C to remove tissue debris. Protein quantification was measured by a BCATM Protein Assay Kit (Pierce). We usually used 50–100 μg of proteins for immunoblotting. The clarified lysates were mixed with 5 × SDS sample buffer (300 mM Tris–HCl, pH 6.8, 10% SDS, 50% glycerol, 5% β-mercapto-ethanol, 0.05% bromophenol blue) and boiled for 5 min at 95°C. Then these protein samples were kept at −20°C for later use.

### 4.6 Immunoblotting

The protein samples were analyzed by western blotting with antibodies against DNAJB13 and beta-actin, respectively. The multi-tissue proteins from adult BALB/c mice and the testes proteins from different stages of BALB/c mice were separated by SDS–PAGE on a 12% polyacrylamide gel and transferred to PVDF membrane (Millipore). Western blotting analysis was performed as described previously [13]. The membranes were blocked with TBST (25 mM Tris–HCl, pH 8.0, 125 mM NaCl, 0.1% Tween 20) containing 5% nonfat dry milk for 4 h at r.t. and incubated with the primary antibodies in blocking buffer overnight at 4°C. After washing with TBST, the blots were incubated with the secondary antibodies in blocking buffer for 1 h at r.t. Both antibodies were conjugated to horseradish peroxidase. The membranes were detected with Lumigen™ PS-3 detection kit (GE Healthcare UK Limited) according to the manufacturer’s directions and exposed to X-ray film (Fuji, Tokyo, Japan). The primary antibodies used in this study were TSARG6 (F-20) goat polyclonal antibody (1:50 dilution) (Santa Cruz), and mouse anti-beta-actin monoclonal antibody (1:1000 dilution) (Sigma). The secondary antibodies were HRP-conjugated donkey anti-goat IgG antibody (1:20000 dilution) (KPL) and HRP-conjugated goat anti-mouse IgG antibody (1:4000 dilution) (KPL).

### 4.7 Immunohistochemistry

Mouse testes were collected from adult BALB/c mice and BALB/c mice at postnatal weeks 0, 1, 2, 3, 4, 5, 6, 7, and 16, and immediately fixed in freshly made 4% paraformaldehyde (PFA) solution for 4 h at r.t. After fixation, tissues were embedded in paraffin wax, and 5–6-μm sections were prepared and kept at 4°C until further use. After deparaffinization and rehydration, the sections were boiled in 0.01 M sodium citrate (pH 6.0) in a microwave oven for 10 min to retrieve the antigens. The following staining procedure was performed as described previously [13]. After a brief rinse in 1 × PBS, the sections were blocked with 5% BSA in 1 × PBS (blocking buffer) for 30 min at r.t and then incubated with the primary antibody diluted in blocking buffer overnight at 4°C. The primary antibodies used in this experiment were TSARG6 (F-20) goat polyclonal antibody (1: 50 dilution) (Santa Cruz), and goat pre-immune sera. After washing in 1 × PBS, the sections were incubated with HRP-conjugated donkey anti-goat IgG antibody (1:20000 dilution) (KPL) diluted in blocking buffer for 1 h at r.t. After counterstaining with hematoxylin, the sections were dehydrated, mounted in resin and examined with an Olympus BX51 microscope (Olympus Corp., Japan). Images were captured using a Hamamatsu ORCA-HR digital camera (Hamamatsu, Japan) with a MicroColor filter operated by CRI RGB Color Merge software (CRI, Inc., Boston, MA, USA).

## Competing interests

The authors declare that they have no competing interests.

## Authors’ contributions

LWN participated in the design of the study, carried out all the experimental procedures and wrote the manuscript. LG conceived and supervised the study, and revised the manuscript. Both authors read and approved the final manuscript.
